# Exploring the Genetic Orchestra of Cancer: The Interplay Between Oncogenes and Tumor-Suppressor Genes

**DOI:** 10.3390/cancers17071082

**Published:** 2025-03-24

**Authors:** Sajal Raj Singh, Rakesh Bhaskar, Shampa Ghosh, Bhuvaneshwar Yarlagadda, Krishna Kumar Singh, Prashant Verma, Sonali Sengupta, Mitko Mladenov, Nikola Hadzi-Petrushev, Radoslav Stojchevski, Jitendra Kumar Sinha, Dimiter Avtanski

**Affiliations:** 1 GloNeuro, Sector 107, Vishwakarma Road, Noida 201301, India; 2 School of Chemical Engineering, Yeungnam University, Gyeongsan-si 38541, Republic of Korea; bhaskar88@yu.ac.kr; 3 Research Institute of Cell Culture, Yeungnam University, Gyeongsan-si 38541, Republic of Korea; 4 Symbiosis Centre for Information Technology (SCIT), Symbiosis International (Deemed University), Rajiv Gandhi InfoTech Park, Hinjawadi, Pune 411057, India; 5 School of Management, BML Munjal University, NH8, Sidhrawali, Gurugram 122413, India; 6 Department of Gastroenterology, All India Institute of Medical Sciences (AIIMS), New Delhi 110029, India; 7 Faculty of Natural Sciences and Mathematics, Institute of Biology, Ss. Cyril and Methodius University, 1000 Skopje, North Macedonia; 8 Friedman Diabetes Institute, Lenox Hill Hospital, Northwell Health, New York, NY 10022, USA; 9 Feinstein Institutes for Medical Research, Manhasset, NY 11030, USA; 10 Donald and Barbara Zucker School of Medicine at Hofstra/Northwell, Hempstead, NY 11549, USA

**Keywords:** oncogenes, tumor-suppressor genes, cancer genetics, carcinogenesis, gene mutations, cell cycle regulation, epigenetics in cancer, molecular pathways, targeted cancer therapy, proto-oncogene proteins

## Abstract

Cancer is complex because of the critical imbalance in genetic regulation as characterized by both the overexpression of oncogenes (OGs), mainly through mutations, amplifications, and translocations, and the inactivation of tumor-suppressor genes (TSGs), which entail the preservation of genomic integrity by inducing apoptosis to counter the malignant growth. Reviewing the intricate molecular interplay between OGs and TSGs draws attention to their cell cycle, apoptosis, and cancer metabolism regulation. In the present review, we discuss seminal discoveries, such as Knudson’s two-hit hypothesis, which framed the field’s understanding of cancer genetics, leading to the next breakthroughs with next-generation sequencing and epigenetic profiling, revealing novel insights into OG and TSG dysregulation with opportunities for targeted therapy. The key pathways, such as MAPK/ERK, PI3K/AKT/mTOR, and Wnt/β-catenin, are presented in the context of tumor progression. Importantly, we further highlighted the advances in therapeutic strategies, including inhibitors of KRAS and MYC and restoration of TSG function, despite which mechanisms of resistance and tumor heterogeneity pose daunting challenges. A high-level understanding of interactions between OG-TSGs forms the basis for effective, personalized cancer treatment—something to strive for in better clinical outcomes. This synthesis should integrate foundational biology with translation and, in this case, contribute to the ongoing effort against cancer.

## Simple Summary

Cancer arises as an abnormality in genomic control, either due to the activation of oncogenes or loss of functioning tumor-suppressor genes. Oncogenes result in an uncontrolled increase in cell proliferation; tumor-suppressor genes act as a brake, leading the cell cycle to stop and promote cell death when necessary. This review seeks to tease out the complex interactions between these genes, throwing light on key signaling pathways such as MAPK/ERK, PI3K/AKT/mTOR, and Wnt/β-catenin, all of which play a role in the progression of the tumor. We discuss major genetic theories, especially Knudson’s two-hit hypothesis, and how modern technologies like next-generation sequencing have advanced our understanding of cancer genetics. We also underscore the most current therapeutic approaches involving oncogenes and the reactivation of tumor-suppressor gene function, with challenges including drug resistance and heterogeneity of the tumor. In this regard, understanding the balance between oncogenes and tumor-suppressor genes is critical in developing effective and personalized cancer treatment, thereby bettering patient outcomes.

## 1. Introduction

Cancer represents a significant health challenge. The Global Cancer Observatory (GLOBOCAN) predictions for 2020 show that 19.3 million cancer cases occurred worldwide [1]. The United States, China, and India are at the top of the list in terms of total cancer cases, reflecting both their large populations and varying risk factors.

**Figure 1:**
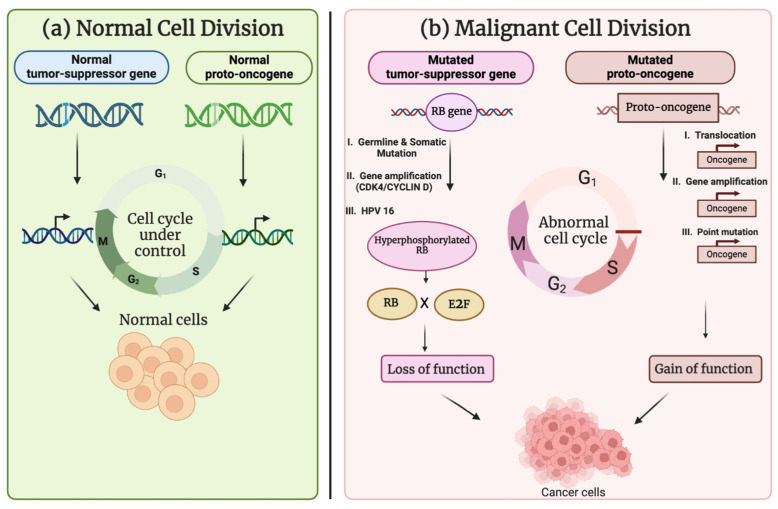
Comparison between normal cell division and malignant cell division. (**a**) With the support of typical proto-OGs and TSGs, a normal cell division preserves tissue homeostasis by self-renewing and supplying the differentiated cell types inside the tissue through a regulated sequence of cell cycles. (**b**) A malignant cell division that causes uncontrolled tumor growth when it undergoes excessive cell divisions due to dysregulated function of mutated proto-OGs and TSGs (created with www.biorender.com).

Keeping cellular growth in control is maintained by the balance in the expression of various proto-oncogenes (proto-OGs) (which can become oncogenes (OGs) when mutated or overexpressed) and tumor-suppressor genes (TSGs) (Figure 1). OGs have the potential to initiate cancer growth by promoting cancer development. Contrarily, TSGs invoice for maintenance of genome stability and pro-modulate cell apoptosis for cells with damaged DNA or activated OGs. These TSGs serve as guardians against the acquisition of loss-of-function mutations and genes that are gatekeepers for mutation. They are important to the cancer process; these damaged cells, however, do not progress toward oncogenesis [2,3].

The most critical understanding of OGs interacting with TSGs was that Alfred Knudson’s clinical study in retinoblastoma patients led to the development of the ‘two-hit hypothesis’—that is, two hits (probably mutations or losses of both alleles of a gene) are required in order for a tumor to develop. All advances in next-generation sequencing (NGS) technologies have broadened the range and extent of possibilities in detecting and characterizing these critical genes in different types of cancers.

With this review, we intend to present an in-depth comprehension of the complex relationship between TSGs and OGs through malignant growth. It will delve into the historical views and basic principles as well as mechanisms of TSG suppression and OG activation, followed by aberrations in these genes that lead to carcinogenesis. Also included will be some clinical implications of this imbalance, including how the understanding of OG-TSG interactions has been used to develop targeted cancer therapies through which some improvements have been observed in the outcomes of patients with different malignancies.

## 2. Historical Perspective

Retroviral research over the last 40 years has uncovered many new OGs and TSGs [4]. As retroviruses can incorporate their genetic information into the host genome, they are a very good tool for the identification of genes whose actions can trigger tumor growth. Examples of retroviral OGs include the *RAS* family genes (*KRAS*, *HRAS*, and *NRAS*), *MYC*, *BCR*-*ABL*, *HER2*, and *BRAF*, while notable TSGs include *RB1*, *TP53*, *BRCA1*, *APC*, and *PTEN* [4,5].

In the 1970s, Dr. Alfred Knudson proposed the ‘two-hit hypothesis’ to explain the genetic basis of retinoblastoma, a rare juvenile eye cancer [6]. He observed that inheriting a mutation in only one copy of the *RB1* gene predisposed individuals to develop a retinoblastoma when there was a second mutation in the other allele. According to this hypothesis, it was proposed that an RB1 mutation inactivating one of the two alleles (i.e., the ‘first hit’) is necessary to develop a predisposition to malignancy but cannot induce tumor formation by itself. These mutations are mostly inherited or can happen during early life. A second mutation or ‘second hit’ occurs later in life and typically inactivates the remaining functional allele, which leads to a complete loss of RB1 function. Arrest of tumor suppression causes unlimited cell proliferation, which can eventually cause malignant evolution. The discovery of RB1 made a revolution in oncology because this changed the conception of tumor genetics [7]. This gene has emerged as a significant guardian of cellular homeostasis, regulating important activities such as cell cycle control, cell differentiation, and apoptosis [8]. RB1 has been quite the subject, after discovery, of characterization and cloning efforts that enriched our understanding of TSGs and mechanisms of retinoblastoma tumorigenesis and also revealed patterns of inheritance in susceptibility to cancer. The two-hit hypothesis is now an expanded version, including many more TSGs associated with various cancers, and provides an insight toward understanding the genetic mechanisms behind carcinogenesis [9].

The discovery of OGs has been facilitated by advancements in quantitative cell biology and molecular methods for investigating tumor viruses. In 1976, J. Michael Bishop and Harold Varmus described the first proto-OG, v-src, in Rous sarcoma virus (RSV), which causes cancer in chickens. The v-src gene was found to be derived from a normal gene, c-src, which has the potential to become an OG when mutated or activated [4]. Since then, many other proto-OGs have been identified, including *KRAS* (Kirsten rat sarcoma viral oncogene homolog) [10,11], *BRAF* (B-raf proto-OG, serine/threonine kinase) [11,12], and *EGFR* (epidermal growth factor receptor) [13].

The identification of these OGs and TSGs over the last 40 years has led to a fundamental change in oncology research and our comprehension of cancer as a genetic disorder. This understanding has contributed substantially to the formulation of novel therapies, revolutionizing cancer treatment approaches [3,4].

## 3. Molecular Factors Driving Oncogenic Activation and Tumor-Suppressor Gene Inactivation

The cell cycle is tightly regulated by a complex network of proteins, including cyclin-dependent kinases (CDKs) and their regulatory subunits, cyclins. These form complexes that control progression through different phases of the cell cycle by regulating gene transcription and protein activity. Dysregulation of this system can lead to uncontrolled cell proliferation, a hallmark of cancer.

This occurs in an OG activation by several mechanisms—such as point mutations, for example, constitutive protein activation, as in *KRAS* mutation; gene amplification overexpression, *HER2* amplification in breast cancer; and chromosomal translocations making fusion proteins, such as *BCR-ABL* in chronic myeloid leukemia. In contrast with OG activation, the TSGs could be inactivated through loss-of-function mutations, deletions, or epigenetic silencing, such as the hypermethylation of promoters. These genetic and epigenetic alterations can affect various cell cycle regulators. Thus, deleting the CDK inhibitor p27 could promote uncontrolled cell cycle progression. Cyclin D1 overexpresses itself in a majority of cancers and is responsible for the excessive proliferation of cells. Further, cyclin E gains or overexpression can also disrupt normal cell cycle control and genomic stability.

### 3.1. Oncogenes

Proto-OGs act mostly as regulatory agents in quite a few basic biological processes in normal cells. They modulate various growth factors, signal transduction events, and nuclear transcription through OGs operating at other levels of signal transduction. Several proto-OGs are found in mammalian and avian genomes that regulate the dynamics of normal cell differentiation and proliferation [14]. Alterations affecting the regulation of proto-OGs, or the structure of their resultant proteins, can manifest as activated OGs in cancer cells. This activation can occur through various mechanisms, including point mutations, chromosomal translocations, gene amplifications, or epigenetic alterations [15]. Once formed, these OGs become drivers of cellular proliferation and are involved in cancer pathogenesis [16].

**Figure 2:**
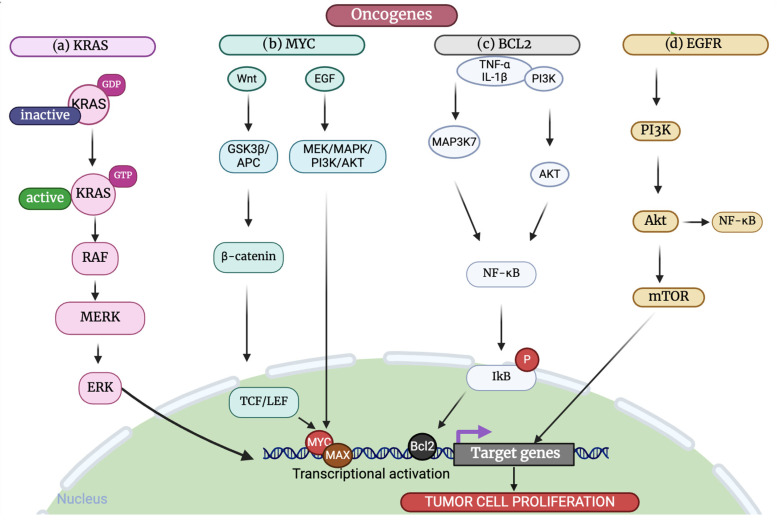
Major oncogenic pathways driving tumor cell proliferation. (**a**) The MAPK pathway is one of the signaling pathways that are triggered by activated KRAS. By switching between an active, GTP-bound state and an inactive, GDP-bound one, KRAS function is regulated. (**b**) When a Wnt ligand attaches to its frizzled receptor, it stops GSK3β from phosphorylating β-catenin and causing it to degrade. As β-catenin builds up in the nucleus, it binds to T-cell factor/lymphoid enhancer factor (TCF/LEF) and activates target genes of Wnt signaling, including MYC. The enhancement of MYC activation due to growth and survival factors, such as EGF, and its downstream mediators, such as MEK/MAPK/PI3K/AKT. (**c**) Activation of NF-κB can be achieved via several routes. A couple of these include PI3K/AKT and MAP3K. Activated MAP3K7 and PI3K/AKT both phosphorylate NF-κB, which, in turn, causes ubiquitination, proteasomal breakdown, and IκB phosphorylation. Released and translocated into the nucleus, an NF-κB dimer attaches to the κB-binding site to upregulate the expression of the Bcl-2 gene. The anti-apoptotic activity is carried out by Bcl-2. (**d**) Growth factor binding to EGFR results in activation of the phosphatidylinositol 3-kinase (PI3K) pathway (PI3K-AKT-mTOR) (created with www.biorender.com).

Such well-documented proto-OGs and their malignant counterparts have been investigated to the greatest depth, including *MYC* as a transcription factor controlling cell proliferation and metabolism, *EGFR* as the receptor tyrosine kinase for signaling pathways of growth and survival in the cell, *KRAS* as a small GTPase activating many signaling cascades, *BRAF* as a serine/threonine kinase of MAPK signaling, as well as *Bcl-2* as the anti-apoptotic protein favoring cell survival. These OGs play integral roles in various cancer types and have been predominantly targeted in cancer therapy (Figure 2).

#### 3.1.1. *MYC*

*MYC* OG, also known as *c-MYC* [17], is a central controller of numerous biological processes and is frequently activated in human cancers. *MYC* regulates fundamental cellular activities involved in intrinsic tumor cell pathways, including growth, differentiation, and metabolism. It primarily functions as a transcription factor, exerting control over the expression of multiple genes, both directly and indirectly [18]. While in non-cancerous cells, *MYC* expression is tightly controlled, and its dysregulation in cancer cells drives proliferation and decreases the apoptosis rate (Table 1) [19]. Genetic changes affecting the *MYC* proto-OG and related signaling pathways are among the most prevalent in human malignancies. *MYC* activation can occur via various genetic, epistatic, epigenetic, and post-translational mechanisms, with nuances observed across different cancer types [17,20]. For example, *MYC* amplification is particularly common in breast cancer, neuroblastoma, and certain types of lung cancer [21,22,23]. Given its widespread involvement in cancer, *MYC* has emerged as an attractive, albeit challenging, target for cancer therapy [24,25,26].

#### 3.1.2. *HER2*

Human epidermal growth factor receptor 2 (HER2), also known as receptor tyrosine-protein kinase erbB-2, is recognized for its function in tyrosine kinase activity. HER2 is a crucial OG, particularly in breast cancer, where its amplification or overexpression occurs in up to 30% of cases, leading to a more aggressive form of the disease [45]. *HER2* overexpression has also been observed in other cancer types, including gastric, ovarian, and non-small cell lung cancers. *HER2* overexpression often leads to its interaction with other members of the EGFR family, forming heterodimers. This results in the phosphorylation of tyrosine residues within the cytoplasmic domain of the heterodimer. Consequently, numerous signaling pathways are activated, promoting cell proliferation and tumor formation [46]. The key components of these cytoplasmic EGFR pathways include STATs, PI-3K/Akt/mTOR, Ras/Raf/MAPK, and PLC-γ/CaMK/PKC [47]. The aberrant activation of specific signaling modules can contribute to tumor growth, metastasis, and resistance to chemotherapy and radiation therapy. HER2-positive cancers are associated with poor prognosis, but the development of HER2-targeted therapies, such as trastuzumab (Herceptin), has significantly improved outcomes for these patients [48,49,50]. However, resistance to these therapies remains a significant challenge, driving ongoing research into novel treatment strategies [46]. The latest investigations have also unraveled the presence of HER2 mutations across a variety of cancer types, whereby HER2 is now understood to play roles other than just that of amplification and overexpression [51].

#### 3.1.3. *KRAS*

The Kirsten rat sarcoma virus oncogene homolog (KRAS) is one of the most well-known OGs, with the highest mutation rate among all OGs. It is linked to several extremely deadly malignancies, such as colorectal cancer (CRC), non-small cell lung carcinoma (NSCLC), and pancreatic ductal adenocarcinoma (PDAC) [52]. Mutations in KRAS correspond to nearly 30–60% of all carrier cases of colorectal cancer, to about 25–30% of pulmonary adenocarcinomas, and it is estimated that there is an alarming figure of around 90% among pancreatic cancers [53,54,55]. When epidermal growth factor (EGF) binds to a tyrosine kinase receptor, such as EGFR, it triggers RAS activation. Upon binding to GTP, activated RAS initiates a series of cytoplasmic signal transduction pathways, including RAF/MEK/ERK, PI3K/AKT, and RAL cascades (Figure 2). Collectively, these pathways regulate cell proliferation, survival, and metabolism. Despite its prevalence and importance in cancer, *KRAS* was long considered ‘undruggable’. However, recent breakthroughs have led to the development of specific inhibitors for the KRAS G12C mutation, offering new hope for targeted therapies (Table 1) [56,57].

#### 3.1.4. *BRAF*

The 766 amino acid BRAF protein is encoded by the proto-OG BRAF. Most RAF proteins have the ability to phosphorylate MEK (MEK1 and MEK2), but BRAF has the greatest capacity for activation. In response to external stimuli, such as hormones, growth factors, cytokines, and environmental stressors, the Ras/Raf/MEK/ERK pathway is activated. This pathway controls cell proliferation, apoptosis, and differentiation [58]. These BRAF mutations are known to alter many transcription factors that are downstream of the BRAF, most of them being regulated by the ERK signaling pathway. In the nucleus, phosphorylated transcription factors activate transcription, leading to an increased expression of genes involving cancer development [59]. The most common BRAF mutation is V600E [60,61,62]. This mutation is particularly prevalent in melanoma (~30–60% of cases) [63], colorectal cancer (~10–15%) [64], and papillary thyroid cancer (~45%) [62]. Mutations in BRAF can affect various downstream transcription factors, many of which are regulated by the ERK signaling pathway. Within the nucleus, phosphorylated transcription factors stimulate transcription, leading to the increased expression of target genes implicated in cancer development [59]. BRAF mutations lead to constitutive activation of the MAPK pathway, resulting in increased cell proliferation, survival, and metastasis [60]. The identification of BRAF mutations has led to the development of targeted therapies, such as vemurafenib and dabrafenib, which have shown significant clinical benefits in BRAF-mutant melanoma and other cancers (Table 1) [65].

#### 3.1.5. *Bcl-2*

The Bcl-2 family of proteins, which includes both pro- and anti-apoptotic members, plays an essential role in regulating apoptosis [66]. Anti-apoptotic members include Bcl-2, Bcl-xL, and Mcl-1, while pro-apoptotic members include Bax, Bak, and BH3-only proteins like Bim and Puma. Normal cells maintain a delicate balance between these regulators to ensure proper cell function and survival. However, disruption of this equilibrium can lead to either excessive cell death or uncontrolled survival, with the latter potentially provoking cancer development [67]. Bcl-2 and other anti-apoptotic family members primarily function by inhibiting the pro-apoptotic Bax and Bak proteins, thereby preventing mitochondrial outer membrane permeabilization and subsequent cell death. Bcl-2, a member of this protein family, was first discovered as an OG in B-cell lymphomas [68]. Since then, numerous studies have shown that alterations in the expression of Bcl-2 proteins, such as Bcl-2 and Bax, are frequently observed in various types of cancer [69,70]. These findings underscore the significant role of Bcl-2 family proteins in tumor development and their potential influence on treatment resistance. It is increasingly evident that targeting Bcl-2 proteins may provide new avenues for cancer therapy [71]. Indeed, the Bcl-2 inhibitor venetoclax has been approved for the treatment of certain leukemias and lymphomas, demonstrating the clinical potential of targeting this pathway [72].

### 3.2. Tumor-Suppressor Genes

Together with OGs, TSGs have a critical role in the regular functioning of cells, controlling vital functions, such as cell division and proliferation, while preventing cancer initiation. Although they are a varied group, all TSGs have one essential characteristic in common: they protect the body from malignant growth. According to the ‘two-hit hypothesis‘, TSG deletion or mutation must inactivate both copies of the gene for cancer to spread. Numerous TSGs are dispersed across the human genome and play roles in the development of various neoplasms [16]. TSGs can be broadly categorized into several types, including ‘gatekeepers’ (e.g., *RB1* and *APC*) that directly regulate cell growth, ‘caretakers’ (e.g., *BRCA1* and *ATM*) that maintain genomic integrity, and ‘landscapers’ that modulate the tumor microenvironment [e.g., *PTEN* and *TGFβ*] [73,74,75].

TSGs influence tumorigenesis through multiple mechanisms. These encompass the regulation of cell cycle progress, encouragement of apoptosis, and inhibition of angiogenesis as well as metastasis. The loss or dysfunctional regulation of such TSGs invokes cascades of molecular events that propel the cell into over-proliferation by developing malignant phenotypes. Besides genetic mutations, epigenetic silencing also takes the form of promoter hypermethylation, which is yet another prominent mechanism of TSG inactivation seen in cancer. It is through halting aberrant cell growth and maintaining genome stability that TSGs take their action. Inactivation of these genes is a major step toward carcinogenic processes, making them highly relevant for any advances in oncology as well as for throwing light on therapeutics development [76]. The restoration of TSG function has emerged as a promising approach in cancer therapy, with strategies including gene therapy, small molecule activators, and targeting downstream pathways [77,78,79].

Various TSGs have been extensively researched, such as RB1, TP53, NF1, and APC, which are elaborated upon further in the following sections.

#### 3.2.1. *TP53*

*TP53*, often referred to as the ‘guardian of the genome’, is one of the most important TSGs. It codes for the p53 protein, which is a very important regulator of cellular homeostasis central to genomic stability and the prevention of tumor formation. In fact, TP53 is responsive to many types of stress, such as DNA damage, activation of OGs, or metabolic stress [80,81]. Under normal physiological conditions, p53 acts as a transcription factor that regulates other genes responsible for mediating cell cycle arrest, apoptosis, and senescence. When DNA or other stress damages the cells, the p53 protein activates itself and induces a cell cycle arrest so that cells may use that time to repair damage. If the damage is too severe, it can initiate the apoptotic pathway. Dangerous cells are eliminated from this process. The third one is cellular senescence, which stops the proliferation of damaged cells [82,83].

Recent studies reveal that, apart from its roles in directing cell fate, p53 manages various immune responses and inflammatory processes, reacting to a variety of cytokine signaling pathways [84,85]. This finding aligns with the understanding that protracted inflammatory conditions trigger stress reactions, which may affect the development and advancement of malignant processes [86].

*TP53* is frequently mutated in human cancers. According to comprehensive genome sequencing studies, *TP53* mutations were present in approximately 42% of cases across various cancer types, as the DNA-binding domain (DBD) is the most frequently altered area in *TP53* [87,88]. Mutations in *TP53* typically fall into two categories: structural mutants and DNA contact surface mutants [87,89]. Structural mutants exhibit diminished protein stability, causing improper folding at normal body temperatures and leading to the loss of DNA-binding capability. On the other hand, DNA contact surface mutants are situated within the DNA-binding region, where mutations obstruct the protein’s ability to bind to DNA. These mutations frequently occur at specific sites, often referred to as ‘hot spot’ mutations of *TP53*, given their high frequency. This distinguishes *TP53* from a large number of conventional OGs, which usually experience nonsense or frameshift mutations that result in the inactivation of shortened proteins.

The prevalence and importance of *TP53* mutations in cancer have made it an attractive target for cancer therapy. Strategies include restoring wild-type p53 function, eliminating mutant p53, and exploiting synthetic lethality [90,91]. Additionally, immunotherapeutic approaches targeting p53 mutants are being explored, leveraging the immune system to recognize and eliminate cancer cells harboring these mutations [92,93,94].

Studies over the last decade have pointed out that *TP53* mutations tend to do more than p53’s traditional tumor-suppressor activities (e.g., cell cycle arrest, DNA repair, senescence, and apoptosis). In a typical loss-of-function (LOF) pattern, missense mutations commonly arise in the DNA-binding domain (amino acid residues 102–292 in humans), generating a protein unable to transcriptionally activate targets like p21 (*CDKN1A*), BAX, or PUMA [95]. As a result, cells acquire more genomic lesions and resist apoptosis. Nevertheless, most of these *TP53* variants, especially those occurring in ‘hotspot’ residues (R175, R248, R273, etc.), also have gain-of-function (GOF) activities. These mutant p53 proteins are capable of inappropriately interacting with transcription factors and co-regulators (e.g., NF-κB and E2F1), eventually leading to oncogenic transcriptional programs [96]. For example, few p53 mutants reprogram cellular metabolism to elevate glucose uptake or glycolytic flux; others promote invasive and metastatic phenotypes by upregulating proteolytic enzymes or modifying cytoskeletal elements [97]. As a result, mutant p53 could be particularly difficult to target in the clinics because treatments must consider not only loss of tumor-suppressive function but also these newly emergent activities that are pro-tumorigenic. Mechanistically, GOF mutant p53 proteins may also aberrantly interact with p63 or p73 and form misfolded protein complexes, sequestering or inhibiting their tumor-suppressing pathways [98]. In some cases, GOF mutants amplify the expression of genes that play roles in cell migration and metastasis, e.g., EGFR, and thereby amplify growth signals. Such reports highlight why mutant p53-specific inhibitors (such as PRIMA-1 analogs) or reactivators (such as APR-246) that could refold certain mutant conformations into near-wild-type conformation are undergoing clinical trials [99].

An equally important level of p53 regulation is through MDM2 and its homologue MDM4 (also referred to as MDMX). MDM2 is an E3 ubiquitin ligase interacting with the N-terminal transactivation domain of p53 and hence marking p53 for proteasomal degradation. During normal homeostasis, p53 transcriptionally activates MDM2, which, in turn, inhibits p53 function, forming an autoregulatory cycle [100]. Yet, amplification or overexpression of the *MDM2* gene is a common occurrence in some sarcomas, gliomas, and breast cancers with otherwise wild-type *TP53* [101]. Downregulation of p53 that follows has a similar effect to LOF, in which tumor cells grow uncontrolled but still possess an intact p53 gene. This situation has led to therapies targeting MDM2, like primarily small-molecule antagonists (e.g., nutlins, idasanutlin, and spiro-oxindoles) that interfere with p53–MDM2 interaction and reactivate endogenous p53 [102]. This reactivation can lead to cell cycle arrest or apoptosis in MDM2-addicted tumors that are reliant on high levels of MDM2 for p53 inhibition. Combination regimens using MDM2 inhibitors in combination with other targeted therapies (e.g., CDK4/6 inhibitors and checkpoint inhibitors) are under active investigation to leverage p53 restoration along with overcoming resistance mechanisms that develop through collateral signaling pathways [103]. Therapeutic strategies need to be tailored to individual *TP53* mutant alleles or to the level of wild-type p53 with high MDM2, which stresses the complexity and clinical importance of p53-targeted therapies.

#### 3.2.2. *RB1*

Retinoblastoma is a rare cancer that affects the retina of infants and young children. It develops when a susceptible retinal cell, thought to be a precursor of cone photoreceptors, acquires mutations in both copies of the retinoblastoma gene (*RB1*). The lack of functional retinoblastoma protein (Rb) leads to unrestrained cellular growth and genetic changes during tumor development [104]. Although Rb is widely expressed in various tissues, cone precursors have unique molecular and biochemical properties that might make them more vulnerable to Rb loss and promote carcinogenesis.

Rb is primarily recognized as a cell cycle regulator, acting by binding to E2F transcription factors to suppress genes associated with cell proliferation [105]. When subjected to mitogenic signals, Rb undergoes hyperphosphorylation by CDKs, thereby alleviating repression and facilitating the transition from the G1 to S phase of the cell cycle [106]. In the absence of mitogenic signals, loss of Rb relieves this repression, allowing cells to proliferate [107]. It is hypothesized that the main role of Rb is to suppress E2F transcription factors and that the loss of this function is a primary driver of retinoblastoma formation [108]. CDK4 and CDK6 activation induces Rb inactivation through phosphorylation. When CDK4 and CDK6 are activated, they phosphorylate Rb, leading to the release of E2F transcription factors. This release allows E2F to promote the transcription of genes required for cell cycle progression, such as those involved in DNA replication and cell division [109]. In the cell cycle, Rb phosphorylation at S807/811 is linked to E2F activity and has been suggested as a marker of Rb hyperphosphorylation [110].

#### 3.2.3. *NF1*

Neurofibromatosis type 1 is a hereditary illness characterized by the formation of benign or, less frequently, malignant tumors on nerves throughout the body that are linked to mutations in the TSG *NF1* [111]. NF1 is essential for the development of new tumors. According to the theory behind TSGs, the initiation of malignant processes requires loss of function in both *NF1* alleles. *NF1* encodes neurofibromin, which is a regulator of many physiological functions, such as cell division, proliferation, and growth. Its ability to inhibit tumors is a result of its negative regulation of the Ras signaling pathway. As a Ras GTPase-activating protein (GAP), neurofibromin helps to hydrolyze Ras-GTP into Ras-GDP, avoiding Ras-mediated signaling cascades linked to cell proliferation and survival [111].

The 60 exons of the *NF1* gene, which, in humans, is located at 17q11.2, have been found to create many alternatively spliced isoforms. Many mutations in *NF1* have been reported; however, it is difficult to identify specific mutations due to the large size and complexity of *NF1*, the lack of mutation hotspots, and the presence of pseudogenes [112]. Most *NF1* mutations found cause a loss of function. These mutations occur in several varieties, including insertions, deletions, and massive deletions, in addition to missense, nonsense, frameshift, and splice-site alterations. These changes may affect nearby genes in addition to the *NF1* gene. Therefore, *NF1* is categorized as a TSG [113].

Loss of function is the most frequent mutation in *NF1* that causes dysregulation in cell growth and proliferation. *NF1* encodes neurofibromin, which functions as a negative regulator of the Ras signaling system regulating biological functions such as cell division, proliferation, and survival. Loss of function of NF1 may cause hyperactivation of the Ras signaling pathway and contribute to carcinogenesis by promoting uncontrolled cell division.

#### 3.2.4. *APC*

It is well-accepted that adenomatous polyposis coli (*APC*) is a TSG that is commonly altered in CRC and that it is essential to the development and progression of colorectal carcinogenesis [114]. *APC* mutational inactivation is a notable early and crucial event specific to the development of CRC. Mutations in the *APC* gene cause shortened gene products, which, in turn, cause abnormal activation of the Wnt signaling pathway and disruption of many other crucial physiological functions. The multifunctional protein encoded by the APC gene is indispensable in maintaining homeostasis in cells via the regulation of various cellular processes, such as adhesion, differentiation, and proliferation. Its tumor-suppressing action is primarily due to its involvement in degrading β-catenin, a core component of the signaling pathway of Wnt [115]. In addition to its role in modulating Wnt signaling, APC has been identified as a versatile regulator of the cytoskeleton, with the capability to interact with and control all three primary components: microtubules, actin filaments, and intermediate filaments. By regulating the cytoskeleton, *APC* contributes significantly to diverse cellular functions, including cell migration, adhesion, polarity establishment, cell division, and morphogenesis [116].

The majority of APC mutations observed in individuals with colorectal cancer produce APC proteins that are shortened at the C-terminus but have an intact N-terminal region. These mutations affect APC cytoskeletal processes and interfere with Wnt signaling. First, shortened APC is unable to correctly localize to establish cell polarity because of the lack of activities related to the microtubule-binding C-terminus. Second, in cancer-associated APC mutations, the incomplete middle domain of the shortened APC leads to unregulated activity of the intact N-terminus, resulting in uncontrolled cell migration and protrusion. While APC-related disturbances in neurology may be more dependent on alterations in cytoskeletal structure, dysregulation of Wnt signaling is a major factor in the development of CRC [117].

## 4. Oncogenes and Tumor-Suppressor Gene Regulation

The regulation of OGs and TSGs is a fundamental aspect of cancer biology that controls basic mechanisms such as cell division, growth, and survival.

### 4.1. Cell Cycle Regulation

Regulation of the cell cycle is a process that ensures the proper division of cells and that the genomes are intact. Therefore, Cyclin-dependent kinases, or CDKs, and their regulatory partners, cyclins, form complexes that drive cell cycle progression. The activation and deactivation of these complexes are regulated by CDK inhibitors known as CKIs: the INK4 family as well as the CIP/KIP family. These CKIs act to prevent tumor formation because they arrest the cell cycle in unfavorable conditions. Dysregulation of the cell cycle is a hallmark of cancer [118]. There may be uncontrolled growth due to the overexpression of cyclins or CDKs, loss of CKIs, or mutation of the regulators of the cell cycle. For example, overexpression of cyclin D1 has been observed in many tumors, including breast and pancreatic cancers [119]. Such approaches have encompassed therapeutic strategies based on cell-cycle dysregulation, such as those involved in the use of CDK4/6 inhibitors, which include palbociclib, ribociclib, and abemaciclib. Such agents can block a cell cycle in the G1 phase in hormone receptor-positive, HER2-negative breast tumors. Insight into the fine mechanisms of cell cycle regulation and altered properties of these mechanisms in cancer should be essential for realizing a selective therapy that would restore normal cell cycle regulation and, hence, tumor growth inhibition [120].

#### 4.1.1. Cyclin D

CDK1–6 has been found to be important in the transition between G1 and S phase when activated by proteolyzed cyclin D. Cyclin D is a proto-OG CCND1, while CDK4 and CDK6 form complexes through interactions with cyclin D and finally result in activation of the mechanism. Dysregulation of the cyclin-CDK complex is one of the characteristics of cancer and is related to changes in angiogenesis, cancer cell migration, apoptosis, senescence, and proliferation [121]. In mammals, there are three variations of cyclin D, cyclin D1, D2, and D3, encoded by *CCND1*, *CCND2*, and *CCND3* genes. Within the particular cell lineages, these three variations are expressed either alone or in combinations. These cyclin D variations are linked to two different CDKs: CDK4 and CDK6. RB and related proteins p107 and p130 are three pocket proteins phosphorylated by the cyclin D-CDK4/6 complex in response to external cues such as cytokines, mitogens, cell–cell contacts, and differentiation inducers. This phosphorylation event triggers cell cycle progression from the G1 to S phase by activating E2F transcription factors, which, in turn, initiate E2F-dependent transcriptional programs.

#### 4.1.2. Cyclin A

Cyclin A protein interacts with CDK1 and CDK2 to form complexes that regulate the transition from the S to G2 phase and from the G2 to M phase of the cell cycle. CDK1 and CDK2 are key regulators of the cell cycle, with CDK2 governing entry into the S phase (DNA replication) and CDK1 controlling exit from the G2 phase (mitosis initiation) [122,123]. CDK2 is inert as a single unit but becomes active by forming a functional heterodimeric complex when it attaches to either cyclin A or cyclin E, which are its regulatory partners. Numerous carcinogenic signaling pathways are modulated by CDK2. Mutations in cyclin in the form of complexes with CDK2 give rise to oncogenic properties [124].

#### 4.1.3. Cyclin B

Cyclin B and CDK1 create a complex that controls the cell cycle’s passage from the G2 to the M phases, which is essential for the start of mitosis [123]. Genomic instability may arise from G2/M checkpoint failure, which may help pave the way for cancer development. As a result, aberrant cyclin B expression promotes uncontrolled cell division, making it easier for cells to become cancerous. Studies have shown that elevated levels of cyclin B significantly affect survival rates in several malignancies, such as non-small cell carcinoma, hepatocellular carcinoma, esophageal squamous cell carcinoma, and breast cancer.

#### 4.1.4. Cyclin E

Dysregulation of the cyclin E/CDK2 complex, a critical hallmark of many human cancers, results from various genetic aberrations. These aberrations collectively trigger oncogenic activation of cyclin E genes (CCNE1 or CCNE2), leading to disturbances in the RB/E2F pathway, increased transcription of cyclin E, and mutations affecting FBXW7 ubiquitin ligase, leading to the pathological accumulation of cyclin E protein and sustained CDK2 activity [125]. The cyclin E/CDK2 complex hyperactivation plays an important role in driving unregulated cell cycle entry from the G1 into the S phase. This promotes genomic instability (a hallmark of cancer). Research studies show that cyclin E overexpression is observed in various types of malignancies, including breast, ovarian, and colorectal cancers [126]. Additionally, it is associated with a poor prognosis and highly aggressive tumor behavior. The contributions of aberrant cyclin E/CDK2 signaling in resistance to therapies reveal an important element that needs to be clearly understood concerning cancer progression [127]. Targeting the cyclin E/CDK2 axis, therefore, offers a promising avenue for therapeutic intervention. The small-molecule inhibitors and potential strategies to restore FBXW7 function hold much promise for treatment modalities that could more elegantly address this fundamental dysregulation in cancer. Activation of the cyclin E/CDK2 complex promotes uncontrolled cell proliferation and malignant transformation, highlighting the cyclin E/CDK2 complex as a promising target for therapeutic intervention in cancer treatment.

## 5. The Interplay Between Oncogenes and Tumor-Suppressor Genes

The progression of cancer can be viewed as a consequence of TSG and OG gene imbalance, whose progression leads to severe disruption of several fundamental biological processes, such as the processes of cell division, growth, and survival. The role of a TSG is to inhibit cell division or to promote cell death when there is a cell strain or when there is considerable damage to DNA, whereas OGs are genes that cause cellular proliferation and differentiation when activated by mutation or overexpression. However, this classification does not provide a complete picture, as both types of genes work together to protect against neoplasia. Oncogenesis happens when both copies of proto-OGs are inactivated [128]. The differentiation and growth of normal cellular phenotypes are primarily regulated by proto-OGs. When mutations occur in proto-OGs, they can transform into OGs, leading to uncontrolled cell division and contributing to cancer. An imbalance in TSGs, such as p53 and RB1, which typically inhibit excessive cell proliferation, can be triggered by OGs or may promote increased cell division. This often results in cancer. For example, mutations in p53 are present in the majority of human tumors and frequently disable their function, allowing malignant cells to proliferate unchecked.

### 5.1. Uncontrolled Cell Proliferation

OGs encourage cell division and proliferation, whereas TSGs function as brakes to prevent uncontrolled cell growth. Tumor development can occur when OGs are hyperactive or when TSGs are dormant.

#### 5.1.1. Inhibition of Apoptosis

TSGs are essential in triggering apoptosis in response to DNA damage or other physiological stressors. TSG inactivation can prevent cell death and the accumulation of mutations that might cause cancer. For example, apoptosis is triggered by proteins of the proto-OGs MYC and BAX, the TSG TP53, and the transcription factor E2F. The oncoproteins BCL2, ABL, and RAS, on the other hand, prevent apoptosis [14].

#### 5.1.2. Genomic Instability

TSGs contribute to the preservation of genomic stability by repairing DNA damage and by halting the accumulation of mutations. TSG loss-of-function mutations can cause genomic instability, which raises the possibility of subsequent mutations and promotes the development of tumors.

#### 5.1.3. Cancer Metabolism

The deletion of RB family members increases glutamine uptake and utilization through E2F-dependent overexpression of ASCT2 and GLS1. When combined, the essential cell division promoters c-Myc and E2F provide cells with access to glutamine, a metabolic substrate necessary for meeting the needs of DNA replication through biosynthesis [129]. Therefore, to avoid such conditions, cancer cells can become constitutive scavengers of accessible glucose, glutamine, and critical amino acids from the extracellular environment due to aberrantly activated OGs and/or loss of TSG function. This allows cancer cells to proliferate in an uncontrolled manner [130].

#### 5.1.4. Epigenetic Modifications

Cancer starts and grows due to genetic and epigenetic events. Epigenetics has an impact on gene expression without changing DNA sequences. This includes DNA methylation, histone changes, and microRNAs. These changes pass down, can be reversed, and may disrupt normal gene function, which leads to cancer. Epigenetic shifts often happen as cancer develops and play a big part in its growth. New findings in epigenetics help to spot cancer markers to assess genetic alterations pertaining to cancer [131].

#### 5.1.5. Long-Non-Coding RNAs

Long non-coding RNAs (lncRNAs) are RNAs longer than 200 nucleotides that are not able to produce proteins. They play a number of functions in the development of human cancer. Growing evidence has demonstrated that lncRNAs participate in invasion and metastasis. A positive feedback loop of KLF5 and long noncoding RNA LINC00152 promotes the growth of breast cancer.

#### 5.1.6. MicroRNAs

MicroRNAs (miRNAs) are a class of small non-coding RNAs (sncRNAs), typically 21 to 24 nucleotides in length, which modulate gene expression by binding to the 3′-UTR region of target mRNAs. This binding can result in the suppression of translation or the cleavage of the mRNA, thereby regulating gene expression. The identification of novel miRNAs has provided fresh perspectives in cancer research. Recent studies have revealed that elevated levels of miR-371-5p result in the downregulation of inhibitor of growth family member 1 (ING1), leading to the enhanced proliferation of pancreatic cancer cells and subsequent tumor growth. It has been shown that NF-kB experiences continuous activation in pancreatic cancer, a process that is controlled by several miRNAs. In particular, it has been found that miR-301a promotes NF-kB activation by suppressing the NF-kB repressing factor (NKRF). Interestingly, miR-301a expression increases in proportion to NF-kB activation, indicating the possibility of a continuous positive feedback loop.

#### 5.1.7. Proto-Oncogene Activation

Research has increasingly supported the idea that proto-OG activation is a critical factor in the development of cancer. This activation can occur through several mechanisms. For example, during retroviral transduction, point mutations, deletions, or gene fusions within the proto-OG coding sequence can lead to abnormal protein products expressed at unusual levels or times [132]. Retroviruses can also alter proto-OG expression via insertional mutagenesis. This process involves the integration of retroviral DNA near or within a proto-OG. The powerful regulatory elements within the retrovirus can influence the proto-OG, leading to improper initiation. Importantly, proto-OG triggering is not caused solely by retroviruses. Other genetic events, such as point mutations and DNA rearrangements (translocations or gene amplifications), can cause abnormal proto-OG activation, resulting in changes in protein expression.

## 6. Signaling Pathways

Signaling pathways form vital networks that control how cells grow, change, survive, and die. In healthy cells, these networks keep things balanced and react to signals from outside. But in cancer, these pathways get mixed up. This leads to cells dividing without control, avoiding death and spreading to other parts of the body. Changes in key parts like receptors, proteins, and gene controllers can turn normal cells into cancer cells. To figure out how cancer starts and gets worse, we need to understand these pathways. This knowledge can also help us to create targeted treatments to fix mixed faulty signaling [133].

### 6.1. MAPK/ERK Pathway

There is a very important relationship between MAPK signaling initiation and the progression of cancer. Often, the MAPK pathway is altered in different types of cancer and plays a role in tumor development, survival, invasion, and metastasis. In mammals, three major signaling pathways of MAPK, ERK, c-Jun NH2-terminal kinase (JNK), and p38 transmit external signals to the nucleus. The ERK kinase family consists of ERK1 (p44) and ERK2 (p42); JNK is formed by three isoforms (JNK1, JNK2, and JNK3), and the p38 MAPK family includes p38a, p38b, p38d, and p38g. It is MAPK that regulates how cells respond to stimuli and, importantly, coordinates a much wider range of biological processes than a few cell divisions and proliferation and the survival of those cells.

Through signal transmission from cell surface receptors, for example, receptor tyrosine kinases (RTKs) and G-protein coupled receptors (GPCRs), the MAPK/ERK pathway is activated. Improper pathway control produces abnormal cellular behavior, such as increased growth and proliferation, dedifferentiation, and survival, which collectively promote the development of cancer [134]. Several receptors are capable of activating the MAPK/ERK signaling pathway, including the fibroblast growth factor receptor (FGFR), platelet-derived growth factor receptor (PDGFR), vascular endothelial growth factor receptor (VEGFR), insulin-like growth factor receptor (IGFR), hepatocyte growth factor receptor (HGFR, also known as c-Met), and stem cell factor receptor (SCFR, also known as KIT). These receptors act as molecular controls, relaying signals from outside the cell to intracellular pathways, activating the MAPK/ERK signaling cascade, and regulating cellular functions, such as differentiation, proliferation, and survival.

The activation of ligands on receptors triggers cytoplasmic tyrosine kinases (TKs) to phosphorylate tyrosine residues, recruiting adapter complexes, such as GRB2/Shc/SOS, to activate the small GTPase protein RAS. Activated RAS recruits the serine/threonine kinase enzyme RAF to the membrane, initiating a cascade that leads to MEK1/2 phosphorylation, ultimately activating ERK1/2. Phosphorylated ERK translocates to the nucleus, where it activates AP-1 transcription factors. These factors induce gene transcription for cell cycle progression and growth factors, thereby establishing an autocrine/paracrine loop. Aberrant MAPK/ERK signaling leads to continuous cell growth and proliferation [134,135].

### 6.2. Wnt/β Signaling Pathway

The Wnt signaling cascade is an essential route for determining cell fate and embryonic patterning regulated by various co-factors, co-receptors, and antagonists. Nineteen of these ligands bind to ten G-protein-coupled frizzled family activity receptors [136]. The β-catenin pathway, sometimes referred to as the canonical Wnt pathway, is an essential signaling cascade that plays a role in essential physiological processes, including embryonic development, tissue homeostasis, and stem cell preservation. This route involves the binding of Wnt ligands to the co-receptor LRP5/6 and Frizzled family of cell surface receptors, which stabilize β-catenin. As a result, stabilized β-catenin builds up and then moves into the nucleus, where it interacts with TCF/LEF transcription factors to trigger the activation of Wnt target genes. Dysregulation of the canonical Wnt signaling pathway has been associated with various developmental and degenerative conditions. Hyperactivation of the Wnt/β-catenin pathway can promote tumor initiation, progression, and metastasis by stimulating cell proliferation, inhibiting apoptosis, enhancing epithelial-to-mesenchymal transition (EMT), and promoting the acquisition of cancer stem cell properties.

### 6.3. PI3K/AKT/mTOR Pathway

Many cancer types typically exhibit dysregulation of the PI3K/Akt/mTOR signaling pathway, which affects multiple processes, including cell survival and proliferation, metabolism, angiogenesis, and metastasis. Numerous upstream signaling proteins regulate this system, which, in turn, interacts with other compensatory signaling pathways. Notably, the RAF/MEK/ERK pathway modifies downstream effectors [137].

PI3K is activated by binding to RTKs on the cell membrane in response to external signals such as growth factors and cytokines. PIP2, or upon activation, PI3K phosphorylates phosphatidylinositol 4,5-bisphosphate (PIP2), converting it into the crucial secondary messenger phosphatidylinositol 3,4,5-triphosphate (PIP3). PIP3 recruits Akt to the plasma membrane, where it is phosphorylated and activated by mTORC2 and phosphoinositide-dependent kinase 1 (PDK1). Activation of the PI3K/Akt signaling pathway can occur through several mechanisms, including amplification of the pathway itself or mutations in key pathway proteins. These mutations can directly activate Akt, or they can inactivate phosphatases such as PTEN and INPP4B, which normally function as tumor suppressors by hydrolyzing PIP3. Additionally, mutations in PIK3CA (encoding the p110a catalytic subunit of PI3K) or PIK3R1 (encoding its regulatory subunit) can lead to pathway hyperactivation. Once activated, PIP3 recruits Akt to the plasma membrane, where it undergoes phosphorylation by PDK1 and mTORC2, leading to its full activation.

### 6.4. p53 Pathway

The TP53 gene encodes the transcription factor p53. Its primary biological role is believed to serve as a TSG and to safeguard the cell’s DNA integrity. TP53 is also involved in cell differentiation, aging, and development [138]. p53 is activated by different cellular stress signals, including DNA damage and OG activation. Many types of cancer, such as colorectal, head and neck, esophageal, lung, pancreatic, and female reproductive cancers (cervical, ovarian, uterine, vaginal, and vulvar) display mutations in the TP53 gene [139].

Under extreme oncogenic stress, activation of the p53 signaling pathway causes senescence and cell-cycle arrest, and this is a critical mechanism controlling the suppression of carcinogenesis. Key signaling nodes involved in the control of mTOR kinase are frequently the site of convergence of oncogenic stimuli. Drosten et al. [140] demonstrated that Ras-dependent and Ras-independent activation of the Raf/MEK/ERK cascade inactivates the p53/p21 complex, hence inducing cell proliferation. These results highlight the significance of p53 inactivation and Ras oncogenic activity in cancer. p53 inhibits the growth and migration of melanoma cells via direct promoter binding and boosting of ITIH5 TSG transcription, most likely via downregulating KLF4 transcriptional activity. The tumor-protective function of TP53 can be compromised by mutations of the gene that frequently occur in hotspots, including R175, G245, R248, R249, R273, and R282. The ubiquitin ligase murine double minute 2 (MDM2) maintains low levels of p53 in normal cells through a negative feedback loop. In this loop, p53 induces MDM2 expression, which, in turn, promotes p53 degradation and inhibits cellular p53 functions.

The rate of p53 gene transcription is lowered by oncogenic signaling pathways that involve STAT3, which binds to the p53 promoter both in vivo and in vitro. A p53 promoter site-specific mutation of a STAT3 DNA-binding site partly reverses STAT3-induced inhibition. A p53 promoter site-specific mutation of a STAT3 DNA-binding site partly reverses STAT3-induced inhibition. Additionally, UV-induced cell growth arrest in normal cells and the p53 response genes are impacted by STAT3 activity. Moreover, blocking STAT3 in cancerous cells raises p53 expression, which causes p53-mediated tumor cell death.

Cooks et al. [141] found that in animal models with mutant p53, mutant p53 and tumor necrosis factor (TNF) prolong NF-κB activation, which leads to the development of colon cancer and a chronic inflammatory phenotype. These findings bolster the idea that NF-κB activation and the accumulation of missense p53 mutations are related to human malignancies associated with colitis. In colorectal carcinomas, the recruitment of RNA polymerase II to specific regulatory elements is modulated by the simultaneous binding of NF-κB, the R273H mutant form of p53, and other mutated variants of p53 to these enhancers. This mechanism enhances mRNA synthesis and triggers the activation of genes that facilitate tumor progression. Consequently, mutant p53 collaborates with NF-κB to alter the inflammatory tumor microenvironment (TME), leading to the upregulation of genes that support cancer growth in various epithelial and non-epithelial cell types.

### 6.5. Notch Pathway

The notch pathway modulates different cell differentiation processes at the embryonic and adult phases of life and has been linked to cancer or disease progression. In 1914, Boveri [142] proposed the somatic mutation theory of carcinogenesis (SMT), which posits that the onset of malignancy begins with the acquisition of a crucial mutation in a cell capable of replication, followed by the successive accumulation of mutations through a series of steps, driving the progression toward malignancy.

## 7. Feedback Loops

### 7.1. Negative Feedback Loops

In cancer research, negative feedback loops may involve various molecular mechanisms aimed at regulating aberrant cell growth, proliferation, and survival. For example, TSGs such as p53 often function through negative feedback loops to control cell cycle progression and induce apoptosis in response to cellular stress or DNA damage. Additionally, negative feedback loops can also operate within signaling pathways implicated in cancer, such as the p53–Mdm2 autoregulatory loop, to prevent uncontrolled cell growth and proliferation.

Recently, it was shown that migration and invasion inhibitory protein (MIIP) inhibits the growth of tumors and forms a negative feedback loop with HIF1a in pancreatic cancer [143]. Normal cellular processes depend on the strict regulation of receptor tyrosine kinase signaling, and unregulated signaling can result in cancer. The receptor tyrosine kinase known as FGFR2 promotes migration and proliferation. Tumor growth is aided by FGFR2 dysregulation, and activating mutations in FGFR2 are seen in a variety of cancer types. A negative feedback loop is created when ERK1/2 phosphorylates S780 in FGFR2. When this feedback loop is disrupted in cancer cells, FGFR2 signaling is hyperactivated, which may lead to more aggressive characteristics [144]. Studies on negative feedback loops are now more limited than those on positive feedback loops.

### 7.2. Positive Feedback Loops

KRAS: signaling frequently participates in positive feedback loops in cancer, which promotes the growth and aggressiveness of tumors. One process is the activation of KRAS, which is usually brought about by signals from upstream sources or growth factor receptors. Upon activation, KRAS triggers downstream signaling cascades that support cell survival, proliferation, and metastasis, including the PI3K/AKT and MAPK signaling pathways [145].

STAT3: since inhibiting STAT3 in tumors with constitutively active STAT3 has been shown to cause cell death, STAT3 is increasingly being studied in relation to anti-cancer treatment [145]. TP53 is transcriptionally inhibited by STAT3; however, TP53 mutations in cancer partially overcome this restriction [146]. The interactions between STAT3 and mutant p53 have been clarified by recent studies. According to one study, mutant p53 maintains STAT3 phosphorylation by displacing the phosphatase SHP2 [147] in contrast to wild-type p53. In a different study, scientists discovered that blocking STAT3 in pancreatic cancer cells disrupts HSP90 and molecules linked to the mevalonate pathway, which, in turn, lowers the amounts of mutant p53. It is interesting to note that STAT3 phosphorylation is mediated by the production of mutant p53. These results highlight the presence of a feedback loop that is mediated by mutant p53 and STAT3, forming an antagonistic partnership that is essential for driving cancer.

## 8. Artificial Intelligence in Cancer Research

**Figure 3:**
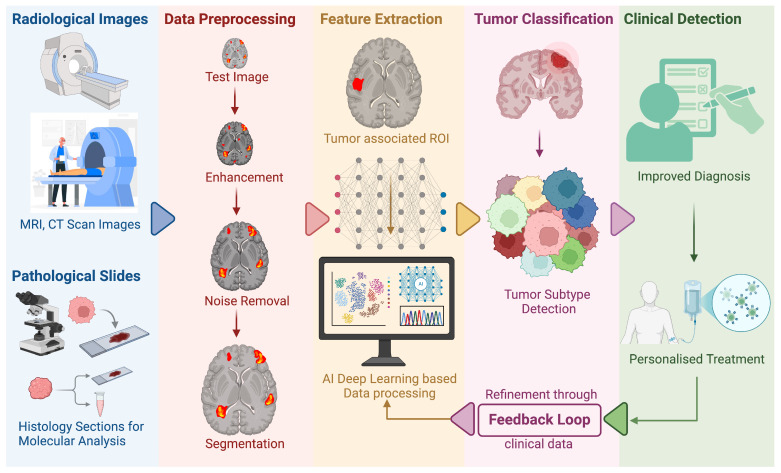
AI-based method for better cancer diagnosis by combining radiological imaging and histopathology examination. Initially, the medical images, such as MRI and CT scans and histology slides, are pre-processed through enhancement, noise removal, and segmentation to enhance data quality. Then, the features are extracted and analyzed by deep learning models to diagnose tumors and also determine subtypes (if any). The AI-derived insights help significantly in guiding clinical decision making and facilitating diagnosis and treatment planning. A feedback mechanism enables constant improvement of the model as per the actual clinical results, making it more accurate and responsive over time (created with www.biorender.com).

Recently, artificial intelligence has evolved drastically to change the understanding of cancer research. It has occurred through the combination of computational algorithms with large-scale biomedical data to generate precise diagnostic, prognostic, and therapeutic information [148]. One of the most exciting opportunities is in multi-omics integration. This is where genomics, transcriptomics, proteomics, and epigenomics data are merged and processed using machine learning to determine important molecular signatures and candidate therapeutic targets that may be outside the scope of traditional methods [149]. By identifying various subtle patterns and then relating them to patient outcomes, these AI-driven models are able to propose insights on oncogene activation, inactivation of tumor suppressors, and other genetic abnormalities (e.g., mutations) that fuel tumor growth progression. In the diagnostic arena, AI-augmented analysis of radiologic images (e.g., CT and MRI) and histopathologic slides has achieved extraordinary accuracy in cancer subtype classification, sometimes even equaling or exceeding expert pathologists [150]. Deep learning models are particularly good at feature extraction, and they can capture individual morphological features, such as nuclear atypia or tissue architectural abnormalities that can predict not just the occurrence of malignancies but also possible mutations, such as *IDH1* (in glioma) or *KRAS* (in colorectal cancer) [151]. Importantly, automated imaging can speed up diagnostic processes, lower inter-observer variability, and uncertainty, and also allow for earlier therapeutic decision-making. Furthermore, computer vision-based digital pathology platforms equipped with convolutional neural networks are now even more able to segment tumor areas, measure immune cell infiltration, and correlate with these results prognostic predictors [152]. Such developments herald more personalized care as physicians gain richer AI-driven insights into the individualized biology of every tumor (Figure 3).

Aside from classification work, AI-assisted drug discovery provides a strong means for discovering new small-molecule inhibitors or biologics that selectively inhibit cancer pathways. Through the use of techniques such as structure-based virtual screening, reinforcement learning, or GANs, scientists can quickly screen chemical libraries or design new compounds in silico prior to experimental validation [153]. This greatly reduces the preclinical stage and saves resources by focusing on the most promising candidates. AI-usage is also improving the whole process by forecasting off-target effects and toxicity profiles, thereby improving safety considerations early on [154]. Another area of innovation that is related is the optimization of clinical trial design. Machine learning algorithms have the ability to stratify patients according to molecular and clinical markers, being able to predict which patient is more likely to be helped by a particular therapy—for instance, a targeted inhibitor or immunotherapy [155]. It enhances trial efficiency and thus accelerates regulatory approval by focusing on cohorts with the greatest potential for responsiveness. In spite of persistent issues and challenges concerning data heterogeneity, algorithmic explainability, and patient privacy, progress in federated learning and strict validation processes is mitigating these issues gradually [156]. AI’s evolving role in oncology, from diagnostics to drug discovery and trial design, is showcasing its impressive ability to reshape cancer treatment. By converging machine learning with clinical and molecular information under one roof, researchers and doctors are moving closer to truly personalized oncology, where drugs are tailored to the tumor’s unique genetic and phenotypic makeup.

## 9. Therapeutic Advances and Challenges

Despite revolutionary advancements in targeted oncology, therapeutic resistance has been a significant setback. Under the selective pressure of targeted drugs, cancer cells can acquire changes that induce disease recurrence and progression [157]. There are a number of mechanisms involved in this effect, including the acquisition of secondary mutations that reverse the drug’s action, activation of alternative signaling pathways, and overall phenotypic changes enabling cells to survive and proliferate despite treatment [158]. For instance, non-small cell lung cancers (NSCLCs) harboring activating EGFR mutations frequently develop the T790M substitution upon prolonged exposure to first- or second-generation EGFR tyrosine kinase inhibitors (TKIs), drastically diminishing drug binding affinity [159]. In anaplastic lymphoma kinase (ALK)-positive lung tumors, the G1202R mutation confers substantial resistance to multiple ALK inhibitors [160]. Striking these resistance mutations with next-generation inhibitors such as osimertinib has been promising, but there are still further adaptations that can arise, underscoring the dynamic and evolutionary nature of cancer. Another central basis for resistance is that of compensatory signaling or pathway bypass, in which parallel pathways become activated subsequent to the inhibition of an initial oncogenic driver [161]. For instance, MET amplification may offer a second growth signal in EGFR-mutant lung cancer treated with EGFR inhibitors [162]. Similarly, RAS/RAF/MEK/ERK feedback loops can reinstate downstream effectors despite the inhibition of MEK or BRAF, making the tumors partially or completely resistant [58]. Strategies co-targeting more than one node in or between pathways (e.g., biallelic PI3K/MEK inhibition in *KRAS*-driven cancers) can abrogate these adaptive processes.

There is also resistance due to phenotypic plasticity and epithelial-to-mesenchymal transition (EMT). EMT confers a stem-like phenotype on cells, altering adhesion molecules, cytoskeleton proteins, and signaling receptors that permit enhanced invasiveness, metastasis, and survival in unfavorable environments [163]. EMT-related transcription factors (e.g., TWIST and SNAIL) can repress epithelial markers and induce pathways that surpass the growth factor receptor blockade. Therefore, the reversal or prevention of EMT via targeted approaches may enhance sensitivity to inhibitors or immunotherapy-based treatments. In addition to the intrinsic characteristics of the cancer cell, the tumor microenvironment (TME) has a significant impact on therapeutic response. TME consists of immune infiltrates, stromal fibroblasts, and the extracellular matrix (ECM). Fibroblast-derived TGF-β can trigger EMT or immunosuppressive niches, whereas cytokines produced by tumor-associated macrophages (TAMs) can shield cancer cells from apoptosis or enhance invasive capabilities [164]. Such immune and stromal cues may override the actions of targeted drugs, making combination regimens to disrupt TME–tumor crosstalk imperative.

Concurrently with such resistance mechanisms, immunotherapy, specifically checkpoint inhibitors directed against PD-1/PD-L1 or CTLA-4, has completely transformed therapy in various cancers [165]. However, few patients show lasting responses due, in part, to immune evasion as well as unfavorable TME conditions [166]. Tumor cells harboring inactivating mutations of the tumor-suppressive genes *TP53* or *PTEN* are able to control antigen presentation and immune system sensitivity [167]. Synergy with immune checkpoint inhibition and with agents that target disruption of oncogenic signaling can potentially be synergistic because inhibition of single drivers (e.g., BRAF and EGFR) makes the tumor cells more immunogenic, while T-cell activation is simultaneously unshackled [168]. Existing clinical trials are also examining CAR-T cell therapies against solid tumors in combination with stroma-modulating drugs to destabilize the protective TME scaffold. Finally, therapeutic resistance can be overcome by monitoring tumor evolution in real-time. Liquid biopsy advances, like sequestering circulating tumor DNA (ctDNA) and circulating tumor cells, enable dynamic measurement of mutational or transcriptomic alterations during therapy. Longitudinal analysis combined with patient-specific biomarker profiling provides a route to personalized combination therapies that target both the genetic drivers and the TME signals underlying resistance. Even with challenges remaining like drug toxicity, optimizing sequencing of combination therapies, and resistance to immunotherapy, research that persists with the aim of preventing adaptive responses and reprogramming the TME remains an ongoing necessary frontier for the long-term control or eradication of cancer.

## 10. Conclusions

Cancer denotes, possibly more than anything else, a relatively heterogeneous disease whose main attributes are variations of modulators in its genetic code, characterized mainly by the overexpression of OGs and inactivation of TSGs. This aberrant behavior provokes uncontrolled cellular proliferation and resistance to apoptosis, which can be regarded as core attributes of malignant growth. These interactions between OGs and TSGs might provoke dysfunction of cellular processes like the cell cycle, apoptosis, or metabolic pathways, all of which are disturbed during cancer. Important insights into the roots of carcinogenesis and cancer development were provided by older concepts, such as Knudson’s two-hit hypothesis, which guided the understanding of the cancer genetics field and provided an entry point into the discovery of a few critical genes involved in tumor initiation and progression. Furthermore, advancements in next-generation sequencing and epigenomic profiling have provided more detailed insights into a number of the novel pathways underlying OG and TSG imbalances.

Tumorigenesis is, in large part, a balance between OGs and TSGs. These genes control important functions, including cell division, growth, and survival. OGs, when mutated or overexpressed, activate cell proliferation and differentiation. When DNA becomes damaged, TSGs act as guardians; they either halt cell proliferation or induce cell death. When such control systems fail, typically as a result of the loss of TSGs or the hyperactivation of OGs, their cells proliferate uncontrollably, and their DNA becomes unstable, showing significant cancer signs.

Essential mediators of cancer progression, such as MAPK/ERK, Wnt/β-catenin, and PI3K/AKT/mTOR pathways, contribute to disease by facilitating cellular proliferation and migration. Difficulty in these pathways due to changes such as mutations or overactivity can trigger the transition to growth and tumor formation. If, for example, there is over-activity of the MAPK/ERK pathway, it can lead to uncontrolled cell proliferation. Mutations in the p53 gene, meanwhile, can allow cells to evade typical rules of growth, never to die. Feedback loops involving proteins like KRAS or STAT3 can also accelerate tumor growth and render treatment more challenging. This illustrates how complex cancer signaling can be.

All the successful attempts to battle cancer are mainly through the principles of tumor biology. Modifying these two strategies involves targeting the tumor microenvironment with anti-fibroblast targeted drugs and ECM resistance to enhance immune responses and immunotherapy efficacy. Oncolytic viruses can activate the immune system due to their dual function and exhibit better results when used in combination therapy with immune checkpoint inhibitors. Nanotechnology is impressive because it allows for precision-targeted therapies with fewer adverse effects from medication delivery and immunotherapy. Radioimmunotherapy has undergone several improvements, with new alpha and beta particle treatments improving cancer-targeting techniques. Artificial intelligence (AI) enables the identification of novel pharmaceuticals and makes clinical trials more tailored and convenient. In conclusion, using metabolic reprogramming as a weapon for ruining energy pathways of cancer cells is another possible therapeutic approach with new therapeutic targets. Consequently, these novelties will enhance the development of more effective, personalized, and targeted cancer therapy.

## Figures and Tables

**Table 1:** This table summarizes different characteristics of important OGs, providing their main function, pathways involved, mode of action, regulations, and clinical significance.

Gene	Main Functions	Mechanism of Action	Signaling Pathways Involved	Role in Normal Development	Regulation	Interactions with Other Genes/Proteins	Protein(s) Structural Features	Frequent Mutations	Associated Cancer Types	Clinical Significance
RAS	Regulates cell growth, proliferation, differentiation, and survival [27]; acts as a molecular switch in various cellular functions [28]	Cycles between active GTP-bound and inactive GDP-bound states [28]; activates downstream effector pathways when in GTP-bound form [27]	MAPK [27], PI3K [29]	Controls signaling pathways regulating cellular functions [27]; involved in growth control, protein biosynthesis, and membrane traffic [28]	Regulated by GTP/GDP exchange factors [28]; controlled by GTPase-activating proteins (GAPs) [28]	Forms complexes with Shc, Grb2, and proline-rich tyrosine kinase [30]; interacts with PI3K and Raf kinases [27]	Contains a GTPase domain functioning as a molecular switch [28]; includes conserved structural core common to GTPase domains [28]	Oncogenic mutations present in almost 25% of human cancers [27]; BRAF V600E mutation associated with RAS signaling in some cancers [31]	Colorectal cancer [29]; papillary thyroid cancer (associated with BRAF V600E mutation) [31]; pancreatic cancer [32]	Crucial target for cancer therapy due to its involvement in multiple signaling pathways [27]; RAS mutations can affect treatment response and prognosis in colorectal cancer [29]
MYC	Modulates transcription of thousands of genes; coordinates cellular processes essential for growth, proliferation, differentiation, self-renewal, and apoptosis [33]	Acts as a transcription factor; forms complexes with other protein to control gene expression	Wnt/β-catenin [34], mTORC1 [34]	Regulates ribosome biogenesis [34]; controls cell identity and disease [35]	Regulated by post-translational modifications, including acetylation and deacetylation [36]; protein stability controlled by PLK1 [33]	Interacts with PLK1, which contributes to MYC protein stabilization [33]; forms complexes with transcription factors and co-activators at super-enhancers [35]	Contains DNA-binding domains and protein–protein interaction regions; subject to post-translational modifications affecting its function and stability	Overexpression of MYC is a hallmark of many human cancers [33]; mutations in MYC regulatory regions can lead to its dysregulation	Multiple myeloma [35]; osteosarcoma [33]; laryngeal cancer [37]	High MYC expression often correlates with poor prognosis in cancer [33]; serves as a potential biomarker and therapeutic target in various cancers
EGFR	Regulates cell growth, proliferation, differentiation, and survival; mediates signal transduction in response to growth factors	Activates upon binding of ligands like EGF; undergoes dimerization and autophosphorylation, triggering downstream signaling cascades	MAPK/ERK, PI3K/AKT, STAT	Crucial for embryonic development and tissue homeostasis; involved in the development of various organs, including the brain	Controlled by ligand availability and receptor internalization; regulated by post-translational modifications and protein–protein interactions	Forms complexes with proteins like Grb2 and SOS; interacts with SRC tyrosine kinase upon activation	Consists of an extracellular ligand-binding domain, a transmembrane domain, and an intracellular tyrosine kinase domain; contains multiple phosphorylation sites in the intracellular domain	Overexpression or activating mutations common in various cancers; EGFR gene amplification observed in some tumors	Non-small cell lung cancer; glioblastoma; head and neck squamous cell carcinoma	EGFR status used as a prognostic and predictive biomarker in cancer; overexpression often correlates with poor prognosis and treatment resistance
HER2	Regulates cell growth, proliferation, differentiation, and survival [38]; plays a role in mammary gland development and breast carcinogenesis [39]	Activates upon dimerization with other HER family members; triggers downstream signaling cascades through autophosphorylation [38]	MAPK/ERK, PI3K/AKT, STAT	Crucial for mammary gland development [39]; involved in embryonic development and tissue homeostasis	Controlled by ligand availability and receptor internalization; regulated by post-translational modifications and protein–protein interactions	Forms complexes with other HER family members; interacts with prolactin receptor (PRLR) signaling pathways [39]	Consists of an extracellular ligand-binding domain, a transmembrane domain, and an intracellular tyrosine kinase domain; exists in full-length, splice variant (d16HER2), and truncated (p95HER2) forms [38]	HER2 somatic mutations occur in about 2% of breast cancers [40]; ERBB2 mutations can lead to trastuzumab resistance [41]	HER2-positive breast cancer (15–20% of breast cancers) [38]; some gastric and gastroesophageal cancers	HER2 overexpression/amplification is associated with poor survival in breast cancer patients [40]; used as a prognostic and predictive biomarker in cancer
BCL-ABL	Promotes cell proliferation and survival; inhibits apoptosis in leukemic cells	Constitutively active tyrosine kinase; activates multiple signaling pathways, promoting cell growth and survival	MAPK/ERK, PI3K/AKT STAT	Not present in normal development; result of chromosomal translocation	Regulated by post-translational modifications; controlled by protein–protein interactions	Interacts with BCL-2, potentially influencing apoptosis regulation [42]; forms complexes with various signaling proteins	Contains an intrinsically disordered region essential for protein function and stability; includes the tyrosine kinase domain from ABL and regulatory domains from BCR	The fusion itself is the primary mutation; secondary mutations can occur, leading to drug resistance	Chronic myeloid leukemia (CML); some cases of acute lymphoblastic leukemia (ALL)	Presence of BCR-ABL is diagnostic for CML; used as a target for monitoring treatment response and disease progression
BRAF	Regulates cell growth, proliferation, and survival; mediates cellular responses to growth signals	Activates the MEK-ERK signaling cascade; phosphorylates downstream targets to promote cell proliferation	MAPK/ERK, PI3K/AKT (indirectly)	Essential for embryonic development; involved in cell differentiation and organ development	Activated by RAS proteins; regulated by phosphorylation and protein–protein interactions	Interacts with MEK1/2, its primary downstream targets; Forms complexes with scaffold proteins like KSR	Contains a kinase domain and regulatory regions; includes an activation segment that regulates kinase activity	V600E mutation accounts for about 90% of BRAF mutations in cancer; other mutations include V600K, V600R, and K601E	Melanoma (40–60% of cases); colorectal cancer (5–10% of cases); papillary thyroid cancer (40–45% of cases)	BRAF mutation status is a prognostic and predictive biomarker in several cancers; used to guide treatment decisions, particularly in melanoma
PIK3CA	Regulates cell growth, proliferation, survival, and migration; generates 3′-phosphoinositides that activate various cellular targets	Catalyzes the production of phosphatidylinositol 3,4,5-trisphosphate (PIP3); activates downstream signaling cascades through PIP3 generation	PI3K/AKT/mTOR, MAPK (indirectly)	Essential for embryonic development; involved in cell differentiation and organ development	Controlled by growth factor receptor tyrosine kinases; regulated by PTEN, a tumor suppressor that counteracts PI3K activity	Interacts with regulatory subunits of PI3K; forms complexes with RAS proteins	Contains a kinase domain and regulatory regions; includes hotspot mutation sites in the helical and kinase domains	Hotspot mutations include E542K, E545K (helical domain), and H1047R (kinase domain) [43]; mutations can lead to constitutive activation of the PI3K pathway	Breast cancer; CRC; thyroid cancer [44]	PIK3CA mutations serve as prognostic and predictive biomarkers in various cancers; mutation status guides treatment decisions, particularly in breast cancer

## Data Availability

No new data were created.

## Abbreviations

The following abbreviations are used in this manuscript:

AI	Artificial intelligence
ALL	Acute lymphoblastic leukemia
APC	Adenomatous polyposis coli
BRAF	B-raf proto-OG, serine/threonine kinase
CDK	Cyclin-dependent kinases
CML	Chronic myeloid leukemia
CRC	Colorectal cancer
DBD	DNA-binding domain
EGF	Epidermal growth factor
EGFR	Epidermal growth factor receptor
EMT	Epithelial-to-mesenchymal transition
FGFR	Fibroblast growth factor receptor
GAP	GTPase-activating protein
GPCR	G-protein coupled receptor
HER2	Human epidermal growth factor receptor 2
HGFR	Hepatocyte growth factor receptor
IGFR	Insulin-like growth factor receptor
ING1	Inhibitor of growth family member 1
JNK	c-Jun NH2-terminal kinase
KRAS	Kirsten rat sarcoma viral oncogene homolog
lncRNA	Long non-coding RNA
MDM2	Murine double minute 2
MIIP	Migration and invasion inhibitory protein
miRNA	MicroRNA
NGS	Next-generation sequencing
NKRF	NF-kB repressing factor
NSCLC	Non-small cell lung carcinoma
OG	Oncogene
PDAC	Pancreatic ductal adenocarcinoma
PDGFR	Platelet-derived growth factor receptor
PDK1	Phosphoinositide-dependent kinase 1
PI3K	Phosphatidylinositol 3-kinase
PIP2	Phosphatidylinositol 4,5-bisphosphate
PIP3	Phosphatidylinositol 3,4,5-trisphosphate
PRLR	Prolactin receptor
Proto-OG	Proto-oncogene
Rb	Retinoblastoma (protein)
*RB1*	Retinoblastoma (gene)
RSV	Rous sarcoma virus
RTK	Receptor tyrosine kinase
SCFR	Stem cell factor receptor
SMT	Somatic mutation theory of carcinogenesis
sncRNA	Small non-coding RNA
TCF/LEF	T-cell factor/lymphoid enhancer factor
TME	Tumor microenvironment
TK	Tyrosine kinase
TSG	Tumor-suppressor gene
VEGF	Vascular endothelial growth factor receptor
